# Increased Sensitivity of Amoeba-Grown *Francisella* Species to Disinfectants

**DOI:** 10.3390/microorganisms8091260

**Published:** 2020-08-20

**Authors:** Maša Knežević, Valentina Marečić, Mateja Ožanič, Nikolina Špoljarić, Ina Kelava, Marija Ćurlin, Yousef Abu Kwaik, Mirna Mihelčić, Marina Šantić

**Affiliations:** 1 Department of Microbiology and Parasitology, University of Rijeka, Faculty of Medicine, 51000 Rijeka, Croatia; masa.knezevic@medri.uniri.hr (M.K.); valentina.marecic@medri.uniri.hr (V.M.); mateja.ozanic@medri.uniri.hr (M.O.); nikolina.spoljaric@gmail.com (N.Š.); ina.kelava@medri.uniri.hr (I.K.); mirna.mihelcic@medri.uniri.hr (M.M.); 2 Department of Histology and Embryology, Faculty of Medicine, University of Zagreb, 10000 Zagreb, Croatia; marija.curlin@mef.hr; 3 Department of Microbiology and Immunology, College of Medicine, University of Louisville, Louisville, KY 40202, USA; yousef.abukwaik@louisville.edu; 4 Center for Predictive Medicine, College of Medicine, University of Louisville, Louisville, KY 40202, USA

**Keywords:** *Acanthamoeba*, *Francisella*, disinfectant, sensitivity

## Abstract

*Francisella tularensis* is a highly infectious, intracellular bacterium and it is the causative agent of tularemia. The bacterium has been isolated from more than 250 species, including protozoa. Previous studies have shown that the growth of *Legionella pneumophila* within the amoeba results in a dramatic increase in the resistance to disinfectants. Since *Francisella* persists in the environment for years, this study investigates whether *Acanthamoeba castellanii*-grown *F. novicida* exhibits an alteration in the resistance to disinfectants. The disinfectants used are didecyldimethylammonium chloride (DDAC) combined with isopropyl alcohol (D1), benzalkonium chloride combined with DDAC and formic acid (D2), and polyhexamethylene biguanide (PHMB, D3). The effect of disinfectants on the bacterial viability is determined by a colony-forming unit (CFU), by transmission electron microscopy (TEM), by fluorescence microscopy, and the damage of the bacterial membrane. Our data has shown that only a one-log_10_ loss in bacterial viability is exhibited upon treatment of agar-grown *Francisella*, while in amoeba-grown *Francisella* there was a three-log_10_ difference with D3. The D1 disinfectant sterilized the bacteria within 10 s. The treatment of agar-grown *F. novicida* with D2 reduces bacterial viability by seven-log_10_ within 10 s and 15 min, respectively. Surprisingly, the treatment of amoeba-grown *F. novicida* with D2 results in a total loss of bacterial viability. In conclusion, *A. castellanii-*grown *F. novicida* is more susceptible to many disinfectants.

## 1. Introduction

*Francisella tularensis* is a Gram-negative coccobacillus and it is the causative agent of the zoonotic disease of tularemia. The most intensively studied species of the genus *Francisella* are *F. tularensis, F. novicida, F. philomiragia, F. hispaniensis* and *F. noatunesis* [1,2]. Additionally, the new species of *Francisella*, isolated mainly from different water environments, have been characterized in recent years [3]. Two subspecies of *F. tularensis: tularensis* (type A) and *holarctica* (type B) cause tularemia in humans. Due to its ability to cause disease upon inhalation, *F. tularensis* is classified as a biothreat agent [4] and studies with virulent strains must be conducted in biosafety-level 3 laboratory facilities. *F. novicida* is often used as a model organism in studying the pathogenesis of tularemia since it causes the same symptoms of the disease in mice as *F. tularensis* [1,2,5]. In addition, it is possible to maintain *F. novicida* under biosafety—level 2 laboratory facilities, making it more convenient.

*F. tularensis* subsp. *tularensis, F. tularensis* subsp. *holarctica*, and *F. novicida* can form biofilms [6], which are important for the persistence of the bacteria in the environment. *F. novicida* is capable of forming a biofilm in vitro by which it can survive the environmental conditions in mud and waterways for a longer period of time [7]. Furthermore, studies have shown that the recommended concentrations of disinfectants are effective against *S. aureus* and *P. aeruginosa* biofilms, which can be common in healthcare facilities [8]. In addition, some environmental bacteria are often taken up by amoeba. *Acanthamoeba* can interact with a wide range of microorganisms such as viruses, algae, yeasts, protists, bacteria including *Legionella, Pseudomonas, Vibrio, Helicobacter, Listeria, Mycobacteria, Escherichia, Shigella, Chlamydia, Klebsiella and Francisella* [9,10,11,12]. Some bacteria are able to survive and multiply in the amoebae, resulting in various phenotypic modulations including enhanced environmental survival, increased virulence and resistance to antibiotics and disinfectants [9]. It has been shown that the amoeba-grown *L. pneumophila* is more infectious than in vitro-grown bacteria in mice [13]. In addition, amoebic trophozoites may protect intracellular bacteria from eradication by disinfectants. *Acanthamoeba polyphaga*-grown *L. pneumophila* exhibit increased resistance to sodium hypochlorite [14]. *F. tularensis* subsp. *tularensis*, *holarctica*, and *F. novicida* can enter and multiply within *A. castellanii* [15], *H. vermiformis* cells [16,17] and amoebal cysts [18], suggesting a role of amoebae as a natural reservoir for *Francisella*. For most bacterial pathogens, it has been proposed that amoebae serve as an environmental reservoir where bacteria develop and refine their virulence strategies to infect mammalian hosts with similar cellular defense mechanisms. However, our previous studies showed no beneficial effect of *F. novicida* grown in *A. castellanii* on bacterial virulence in mice, which might result from differing life cycles and virulence strategies in macrophages and amoebae [15].

*F. tularensis* can be inactivated by a variety of disinfectants and chemicals including: paraformaldehyde (PFA) [19], formaldehyde and glutaraldehyde [20]. The inactivation can also be conducted by physiological techniques such as heat treatment and ultraviolet radiation [21]. In one study, *F. tularensis* subsp. *tularensis* SCHU P9 was killed by heat treatment (94 °C for 3 min and 56 °C for 30 min), 70% ethanol, methanol, acetone, and 4% PFA [22]. O’Connell et al. reported the killing of *F. tularensis* by routine concentrations of free available chlorine (FAC), one of the most commonly used drinking water disinfectants [23]. FAC, sodium hypochlorite in phosphate buffer, killed *F. tularensis* in drinking water at 0.5 mg/L depending on the water pH and temperature [23].

The disinfectants used in this study are widely used in hospitals and medical facilities to prevent and to control acquired infections. For an adequate disinfection, the manufacturers recommended using the following concentrations of disinfectants: 5% didecyldimethylammonium chloride (DDAC) with isopropyl alcohol, 1% benzalkonium chloride with DDAC and formic acid and 0.2% PHMB for at least 15 min.

One of the primary goals of this study was to determine whether amoeba-grown *F. novicida* exhibits alteration in its sensitivity to disinfection, when compared to in vitro-grown bacteria.

## 2. Materials and Methods

### 2.1. Bacteria and Amoeba Cultures

*F. novicida* strain U112 was grown on buffered-charcoal yeast extract (BCYE) agar plates at 35 ± 2 °C for 48 h. *F. novicida* organisms were harvested from a BCYE plate and suspended in 10 mL phosphate-buffered saline (PBS), before measurement by spectrophotometry to obtain a concentration of 10^9^ colony-forming units (CFU)/mL. *A. castellanii* was obtained from the American Type Culture Collection, 30234. The amebae were grown in the medium 30234 at 25 °C. For the preparation of the inoculums, *A. castellanii* was collected from the culture flasks, centrifuged (350× *g*, 30 min), resuspended in PBS, counted in a hemocytometer (Neubauer chamber), washed once in PBS and suspended in PBS at 10^5^ cells per mL.

### 2.2. Disinfectants

The active compounds of disinfectant one (D1) were didecyldimethylammonium chloride (DDAC) and isopropyl alcohol, of disinfectant two (D2) were benzalkonium chloride, DDAC and formic acid and D3 contained polyhexamethylene biguanide (PHMB). The concentrations of disinfectants used in experiments were as follows: D1: 5%, D2: 0.5, and 1% and D3: 0.2, and 0.5%. All the solutions were prepared from reagent grade chemicals in 100 mL sterile tap water.

### 2.3. Infection of A. castellanii with F. novicida

For the preparation of *F. novicida*-infected *A. castellanii* confluent monolayers of *A. castellanii* were inoculated with *F. novicida* at a multiplicity of infection of (MOI) 10 and incubated for 6 h at 27 °C. After 6 h, the monolayers were washed three times with ATCC glucose-free media to remove the extracellular bacteria and were incubated in fresh ATCC media for 48 h at 27 °C. To plate the intracellular bacteria after culturing in amoeba, the amoeba was lysed with Triton × 100 (0.1%) for 10 min and the intracellular bacteria were plated on BCYE agar for 48 h at 37 °C. *F. novicida* cultured in amoeba were harvested from BCYE plate as described above and the 10^9^ CFU/mL of the bacterial suspension were used in the study.

### 2.4. Fluorescence Microscopy

The antimicrobial activity of the tested disinfectants was evaluated by the Live/dead viability assay (BD™ Cell Viability Kit, Becton, Dickinson and Company, BD Biosciences, San Jose, CA, USA). Briefly, 100 µL of bacterial suspensions (OD = 1) were mixed with 100 µL of different disinfectants and incubated at room temperature for 5 min. The appropriate volume of the bacterial suspension was stained with PI (propidium iodide) and DAPI (4′-6-diamidino-2-phenylindole), incubated with PI for 20 min and with DAPI for 3 min at room temperature in the dark. Fluorescence images were taken on the fluorescence microscope (Olympus BX51, Hamburg, Germany).

### 2.5. Treatment of Bacterial Suspension with Disinfectants

The disinfectant efficacy studies were performed on the agar-grown or amoeba-grown *F. novicida* bacterial suspensions. An equal volume of the bacterial suspension and the disinfectants (0.5 mL) were incubated in different time frames (10 s, 1, 5, 10 and 15 min) at room temperature (RT) in order to study the disinfectant antimicrobial activity on *F. novicida*. The CFU of the bacterial suspension was determined by plating 100 µL of suspensions on BCYE agar plates.

### 2.6. Transmission Electron Microscopy

Transmission electron microscopy analyses were performed to evaluate the effects of the different concentrations of the tested disinfectants on the morphology and the structure of the bacteria. In addition, the size and the shape from the amoeba- and/ or plate-grown *F. novicida* were compared. The bacteria were prepared for TEM by negative staining. An amount of 10 µL of bacterial suspension was applied to the Carbon Coated 200 mesh Cooper Grid (SPI Supplies, West Chester, PA, USA) for 2 min, and drained off from the edge of the grid with filter paper. After that, the grid was stained using 10 µL of 2% phosphotungsticacid for 1 min and drained again with the filter paper. The grid was placed directly into the grid box and allowed to air dry before the observation. Ten fields for each sample were randomly photographed on a TEM (Zeiss 902A).

### 2.7. Leakage of Proteins from Treated Bacterial Cells

In vitro-grown and amoeba-grown *F. novicida* were treated with 5% D1 for 1, 5, and 10 min. At the desired time point, the suspensions were centrifuged at 2700 *g* for 2 min. After the centrifugation, the protein leakage from the treated bacteria was determined by measuring the absorbance values of the cell supernatants using a spectrophotometer at OD 280 nm. The absorbance values of the bacteria supernatants were calibrated with the same disinfectant. The cell supernatants of untreated bacterial suspensions were used as control.

### 2.8. Statistics

Statistical analyses were performed with a GraphPad Prizm version 5.0 software. The degree of significance was defined by using Student *t*-test. * *p* < 0.05 and ** *p* < 0.001 were accepted as significantly different.

## 3. Results

### 3.1. Viability of F. novicida after Treatment with Disinfectants

In this study, we first examined the efficacy of three different disinfectants with different active compounds among disinfectants on *F. novicida* that was grown only on agar after treatment for 10 s, 1, 5, 10 and/or 15 min. The concentration has been chosen based upon the manufacturer’s recommendation. The initial concentration of the bacterium was 1 × 10^9^ CFU/mL.

**Figure 1:**
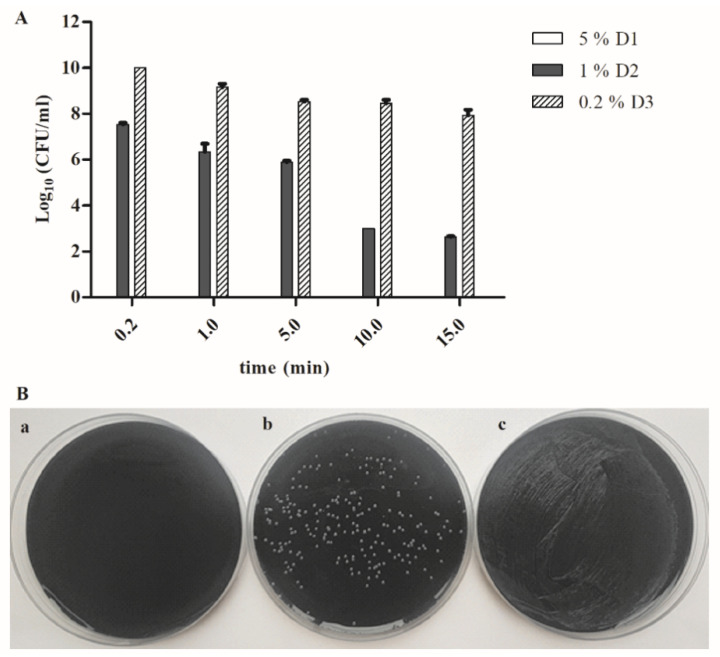
(**A**): The bacterial viability of agar-grown *F. novicida* treated with 5% disinfectant 1 (D1), 1% disinfectant 2 (D2) and 0.2% disinfectant 3 (D3) for 10 s, 1, 5, 10 and 15 min; (**B**): representative pictures of the colony count assay of agar-grown *F. novicida* treated with 5% D1 (**a**), 1% D2 (**b**) and 0.2% D3 (**c**) for 1 min. The bacterial suspension of *F. novicida* (10^9^ colony-forming units (CFU)/mL) was treated with the disinfectant in a 1:1 ratio during 10 s, 1, 5, 10 and/or 15 min. The numbers of the bacteria for each exposure time were determined by CFU on buffered-charcoal yeast extract (BCYE) agar plates. The results are representative of three independent experiments. The experiments were done in triplicate, and error bars represent standard deviations.

Our results show that a 5% DDAC and isopropyl alcohol (D1) disinfectant solution was very efficient in killing *F. novicida* grown only on an agar plate as early as 10 s. Consequently, the total loss of viability occurred at 1, 5, 10 and 15 min (Figure 1A,B).

Furthermore, the number of the bacteria exponentially decreased over time, after the treatment with 1% benzalkonium chloride, DDAC and formic acid (D2), from 3.5 × 10^7^ CFU/mL (10 s), 3 × 10^6^ CFU/mL (1 min), 8 × 10^5^ CFU/mL (5 min), 1 × 10^3^ CFU/mL (10 min) to 4.3 × 10^2^ CFU/mL (15 min) (Figure 1A,B).

Our results showed that ten seconds after the treatment with the 0.2% PHMB (D3) disinfectant, the number of the bacteria increased to 1 × 10^10^ CFU/mL. However, the number of the viable bacteria was gradually reduced to 10^8^ CFU/mL, 15 min after treatment (Figure 1A,B).

We can conclude that 5% D1 disinfectant, which includes active compounds such as DDAC and isopropyl alcohol, has the best bactericidal effect on in vitro-grown *F. novicida* since all bacteria were killed after 10 s. The 1% D2 with active compounds benzalkonium chloride, DDAC and formic acid reduced the bacterial viability from the initial concentration of 1 × 10^9^ to 4 × 10^2^ CFU/mL after 15-min treatment. However, the 0.2% D3 disinfectant—PHMB, reduced the 1-log_10_ bacterial viability after a 15-min treatment.

**Figure 2:**
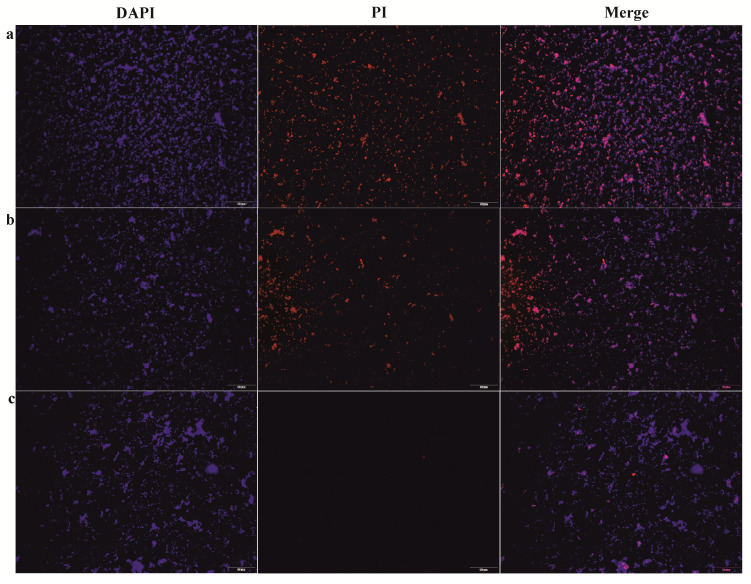
Live/dead viability assay. DAPI/PI staining of *F. novicida* treated with (**a**): 5% D1, (**b**): 1% D2, (**c**): 0.2% D3 for 5 min. Bacterial suspensions (100 µL) (OD = 1) were treated with 100 µL of different disinfectants, incubated at room temperature for 5 min and stained with PI and DAPI. The images were taken on a fluorescence microscope. Ten fields for each sample were randomly photographed.

The live/dead bacterial staining assay was performed as any other control independent assay of bacterial viability (Figure 2). Our results show that all cells exhibited blue (DAPI) fluorescence due to DNA staining regarding dye uptake through the intact membrane of live bacterial cells, whereas the dead bacterial cells displayed red fluorescence (PI) due to the cell membrane permeability after the damage. In relation to the results above, the red fluorescence was the strongest after the treatment with the 5% D1 when 100% of the cells were stained in red. The red fluorescence intensity decreased (56%) after the treatment with 1% D2, while it became dramatically weak (11%) following the 0.2% D3 treatment (Figure 2). These findings are consistent with our determination of viability by CFU.

### 3.2. Loss of Viability of F. novicida Grown in A. castellanii after Treatment with Disinfectants

The previous study has shown that *Legionella pneumophila* exhibits an increased resistance to sodium hypochlorite after being grown in amoeba [14]. Since *Francisella* can survive and replicate in amoeba cells, it is of great interest to determine the viability of the bacteria grown in the amoeba after the treatment with different disinfectants. The statistical significance was determined by comparing the viability of the bacteria grown in *A. castellanii* and agar-grown *F. novicida* after the treatment with disinfectants.

The results of this study show that even ten seconds after the treatment with the 5% D1 solution, *F. novicida* grown in the amoeba were efficiently killed with a total loss of viability. Sequentially, the same results were obtained after a treatment with lower (2% and 1%) concentrations of the D1 disinfectant regardless of the exposure time (data not shown).

**Figure 3:**
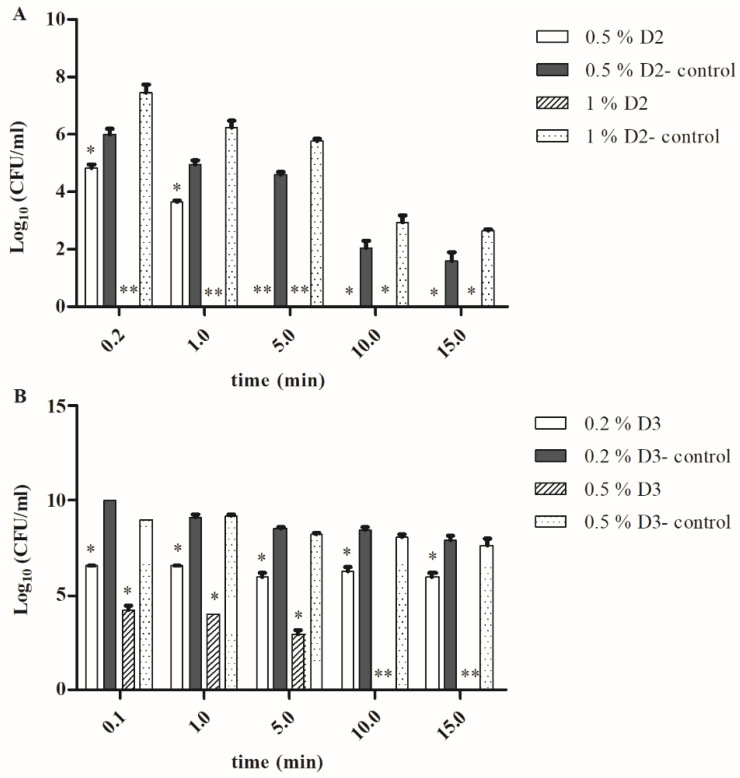
The bacterial viability of *F. novicida* grown in *A. castellanii* after treatment with (**A**): 0.5 and 1% D2. (**B**): 0.2 and 0.5% D3 for different time frames. *A. castellanii* was infected with *F. novicida* at a MOI of 10. After the incubation in amoeba, the intracellular bacteria were plated on a BCYE agar. The bacterial suspension (10^9^ CFU/mL) of *F. novicida* grown in the amoeba was treated with 0.5 and 1% D2 or 0.2 and 0.5% D3 in a 1:1 ratio over different time periods: 10 s, 1, 5, 10 and 15 min. The numbers of the bacteria for each exposure time were determined by CFU on BCYE agar and compared with the control samples. The agar-grown *F. novicida* suspension treated with D2 or D3 solution was used as control. The results are representative of three independent experiments. The experiments were done in triplicate and error bars represent standard deviations. Student *t*-test, * *p* < 0.05, ** *p* < 0.001 were accepted as significantly different from control sample.

Surprisingly, the treatment of the *A. castellanii*-grown bacteria with 1% D2 solution in all tested periods (10 s, 1, 5, 10 and 15 min) resulted in a total bacterial killing. This was significantly different (Student *t*-test, *p* < 0.001 or *p* < 0.05) in comparison to the control samples (bacteria grown only on agar) where the number of the bacteria was 3.5 × 10^7^ CFU/mL after treatment for 10 s with 1% D2. The number of agar-grown bacteria was reduced to 4.3 × 10^2^ CFU/mL after 15 min of treatment with the 1% D2 disinfectant (Student *t*-test, *p* < 0.05) (Figure 3A). Similar results were obtained when a 0.5% concentration of disinfectant D2 was used (Figure 3A).

The treatment of the amoeba-grown *F. novicida* with the 0.2% D3 only reduced the number of the viable bacteria to 1 × 10^6^ after 15 min (Figure 3B). However, this was significantly different in comparison to the bacteria grown on an agar (Student *t*-test, *p* < 0.05), where the 15-min treatment resulted in the 1 × 10^8^ CFU/mL bacterial cells, while the treatment for 5 min resulted 3 × 10^8^ CFU/mL of viable bacteria (Figure 3B). When we increased the concentration of disinfectant D3 to 0.5%, the number of viable bacteria in the control samples slightly reduced to 1 × 10^8^ CFU/mL (15 min), 2.5 × 10^8^ CFU/mL (10 min), 3.3 × 10^8^ CFU/mL (5 min), and 1.5 × 10^9^ CFU/mL (10 s). This was significantly different in comparison to the bacteria isolated from the amoeba where no viable bacteria were detected after a 15- and 10-min treatment with 0.5% D3 disinfectants (Student *t*-test, *p* < 0.01).

The results of this study demonstrated that *A. castellanii*-grown *F. novicida* becomes more susceptible to D2 and D3 disinfectants regardless of the concentration, compared to in vitro-grown bacteria. Our results have also shown the most efficient disinfectant activity of DDAC in combination with isopropyl alcohol (D1). The benzalkonium chloride in combination with DDAC and formic acid (D2) had a better disinfectant effect than PHMB (D3) on in vitro-grown and *A. castellanii*-grown *F. novicida* at the same concentrations.

### 3.3. Membrane Damage

**Figure 4:**
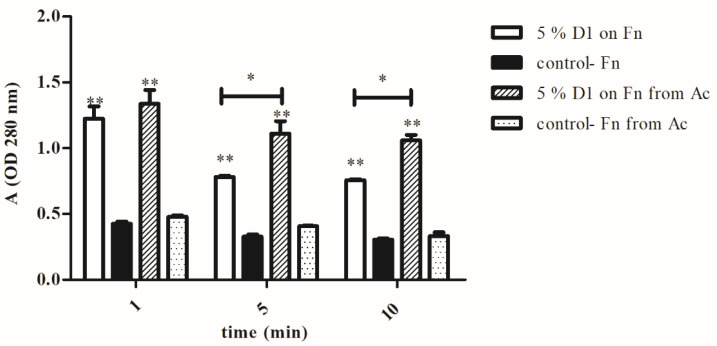
Protein leakage from *F. novicida* (Fn) and *F. novicida* isolated from *A. castellanii* (*Fn* from Ac), treated with 5% D1. The bacteria were treated with 5% D1 for 1, 5 or 10 min. The cells were centrifuged at 2700× *g* for 2 min and the proteins leaked in supernatants were measured as absorbance values at 280 nm. The supernatants of the untreated amoeba- or agar-grown bacteria were used as control. The experiments were done in triplicate and error bars represent standard deviations. Student *t*-test, * *p* < 0.05, ** *p* < 0.001 were accepted as significantly different from control sample.

The release of the cytoplasmic content from in vitro-grown *F. novicida* and *A. castellanii*-grown *F. novicida* after the treatment with the disinfectants was detected as a protein leakage due to the loss of the membrane integrity. The proteins leaked in the supernatants were measured as absorbance values at 280 nm. The protein leakage from the bacterial cells was measured for the D1 disinfectant since it showed the best antimicrobial activity. The agar-grown bacteria treated with 5% D1 for 1 min had significantly higher absorbance value (A = 1.2, Student *t*-test, *p* < 0.001) comparing with the absorbance value of agar-grown untreated bacteria (A = 0.4, Figure 4). In addition, *A. castellanii-*grown bacteria treated with 5% D1 for 1 min had a significantly higher absorbance (A = 1.3, Student *t*-test, *p* < 0.001) than amoeba-grown untreated bacteria (A = 0.4). The absorbance of the amoeba-grown bacteria treated with 5% D1 for 5 min was significantly different (A = 1.1, Student *t*-test, *p* < 0.05) than the absorbance value of the agar-grown bacteria treated under the same conditions (A = 0.7). A similar statistically significant difference was obtained after a treatment with 5% D1 for 10 min (Student *t*-test, *p* < 0.05), where absorbance values of the amoeba and the agar-grown bacteria were 1.0 and 0.7, respectively (Figure 4). The protein leakage from the amoeba-grown bacteria treated with 5% D1 was higher than the protein leakage from the agar-grown bacteria treated under the same conditions (Student *t*-test, *p* < 0.05). Our results also showed that in both in vitro-grown and *A. castellanii-grown F. novicida* the absorbance values at 280 nm were the highest after 1 min of exposure to different disinfectants. After the treatment of the bacterial cells with the disinfectants for 5 and 10 min, we observed the lower absorbance values (Student *t*-test, *p* < 0.05, Figure 4). We can conclude that bacterial killing can be linked to the release of the protein content from vulnerable bacterial cells.

### 3.4. Cell Morphology

**Figure 5:**
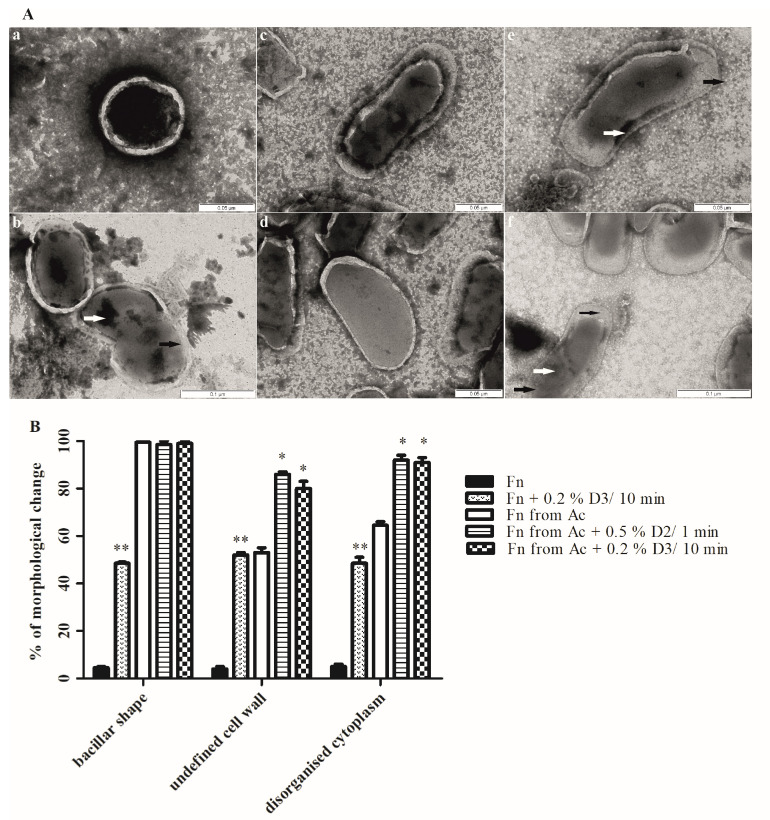
(**A**): TEM images of *F. novicida* (**a**), *F. novicida* treated with 0.2% D3 for 10 min (**b**), *F. novicida* after growth within *A. castellanii* (**c**,**d**), *F. novicida* after growth within *A. castellanii* treated with 0.2% D3 for 10 min (**e**), *F. novicida* after growth within *A. castellanii* treated with 0.5% D2 for 1 min (**f**). The bacteria were prepared for TEM by negative staining. The cell morphology was observed on samples in which around 50% of the bacteria were destroyed by disinfectants. Untreated amoeba-grown and agar-grown bacteria cells were used as control. The ten fields for each sample were randomly photographed. The thick black arrows show an undefined cell wall, the thick white arrows show clumping of the chromatin, thin black arrow shows separation of the cytoplasm from the cell wall. (**B**): Quantitative analyses of the morphological changes and membrane integrity of *F. novicida* (Fn) and *F. novicida* grown in amoeba (Fn from Ac) after treatment with disinfectants. Morphological changes were determined by electron microscopy counting at least 100 bacteria for each sample and using following criteria: (a) bacillar shape, (b) undefined cell wall, (c) disorganised cytoplasm. Student *t*-test, * *p* < 0.05, ** *p* < 0.001 were accepted as significantly different from control sample.

The effect of disinfectants on the morphological changes, the membrane integrity, the size and the shape of agar- and amoeba-grown *F. novicida* was observed using TEM (Figure 5A,B). Since the bacterial viability was not observed with the D1 disinfectant, the concentration of 0.5% for D2 and 0.2% for D3 were chosen to investigate the morphological changes of the bacteria including shape, the integrity of the cell wall as well as the structure of the cytoplasm as a criteria for observation.

In the absence of the disinfectants, 95% of *F. novicida* cells were coccoid shaped with a smooth and intact cell wall. In addition, the cells showed a compact and high electron dense cytoplasm (Figure 5(Aa)). However, changes in the bacterial morphology can be observed following the treatment of the bacteria with the 0.2% D3 (Figure 5(Ab)). The treated bacteria showed a coccobacillar shape and around 50% of the bacteria had an intact cell wall (Student *t*-test, *p* < 0.001). The damage of chromatin was also observed in the treated bacteria in comparison to the untreated bacteria (Student *t*-test, *p* < 0.001).

Interestingly, in comparison to the agar-grown bacteria, *A. castellanii*-grown *Francisella* showed a significant morphological change (Figure 5(Ac,d)). The shape of the bacteria changed to bacillar, the cell wall was highly damaged and the cytoplasm showed intermediate electron density (Figure 5(Ac,d)).

Finally, the bacteria grown in the amoeba were treated with the 0.2% D3 for 10 min and the 0.5% D2 for 1 min (Figure 5(Ae,f)). After the treatment, the bacteria showed significant changes in the morphology compared with the untreated bacterial cells and agar-grown bacterial cells. The highly undefined cell wall was observed in around 80% of the bacteria (Student *t*-test, *p* < 0.05) and the disorganized cytoplasm, with the tendency of clumping in around 90% of bacteria (Student *t*-test, *p* < 0.05). The disinfectant treatment of *F. novicida* grown in amoeba caused the separation of the cytoplasm from the cell wall and the formation of spaces within the cells (Figure 5(Af)).

We conclude that the bacteria morphology changed after growing in the amoeba. The structural damages were observed on the agar-grown bacteria after the treatment with the D3 disinfectant, and highly pronounced in bacterial cells after growing in the amoeba followed by a treatment with 0.5% D2 and 0.2% D3 disinfectant.

## 4. Discussion

The interaction between *F. tularensis* subsp. *holarctica* and *F. novicida* with *A. castellanii* indicates that the amoebae might be an important environmental reservoir for the *Francisella* species [18,24,25]. The virulent strains of the *F. tularensis* type A survive in *A. castellanii* cysts for 3 weeks postinfection and a rapid amoeba encystment is essential for the survival of the bacterium [18].

The replication of *F. novicida* within the amoeba cells is intra-vacuolar and it is very different from mammalian cells, where the cytosolic location of the bacteria is a key aspect in the productive intracellular replication [17]. The ability of *L. pneumophila* to enter and survive within *A. castellanii* has been well characterized. In addition, *L. pneumophila* growth in amoeba has been shown to enhance the ability of the bacteria to survive and replicate in host macrophages and to enhance the virulence in mice [13,26]. *L. pneumophila* can survive within *Acanthamoeba* cysts wherein it is more resistant to the action of biocides [27]. Within an embedded community in amoebas, the disinfectant’s access to the bacteria might be prevented. Surprisingly, in our study, *F. novicida* grown in amoebae were more sensitive to disinfection by the benzalkonium chloride, DDAC and formic acid, and the PHMB. The treatment of th *F. novicida* grown in the amoebae with benzalkonium chloride, DDAC and formic acid resulted in a complete inhibition of the bacterial growth regardless of the exposure time, while the same treatment on the agar-grown *F. novicida* only reduced the number of bacterial colonies in all periods. It would be of great interest to investigate the background of the difference in the resistance of in vitro-grown and amoeba-grown *F. novicida* to the tested disinfectants. Discovering the mechanism of resistance can lead us to better decontamination strategies in the future. A more complete understanding of the decontamination principles can be achieved using different treatments such as heat treatment, ultraviolet radiation, or sodium hypochlorite on *F. novicida* and *Francisella* species that infect humans.

Our results show a 100% efficacy of the 5% DDAC combined with isopropyl alcohol on agar-grown and amoeba-grown *F. novicida*. In all tested intervals, there was no increase in the bacterial colonies, which can be attributed to the sensitivity on the bactericidal action of the disinfectant active substances DDAC and isopropyl alcohol. DDAC is a Quaternary Ammonium Compound (QAC) disinfectant often used in the industry to disinfect hard surfaces because of its relatively low toxicity, broad antimicrobial spectrum, non-volatility, and chemical stability [28]. In a previous study, the disinfectant DDAC showed the bactericidal activity against *L. pneumophila* at concentrations used in cooling tower treatments [29]. In addition, the DDAC is a membrane-active agent and causes membrane leakage of intracellular material [30]. *Staphylococcus aureus* treated with DDAC revealed “bleb” formations on the cell walls as well as morphological and structural changes [30]. Yoshimatsu and Hiyama made an observation with *E. coli* cells where “bleb” formation was also followed by the leakage of intracellular molecules [31]. Chojecka et al. demonstrated that the adaptive resistance of *Pseudomonas aeruginosa* strains can be abolished by using increased concentrations and/or extended contact time of the DDAC in 2-Propanol [32].

In this study, the treatment with the benzalkonium chloride combined with the DDAC and formic acid (D2) and PHMB (D3) was concentration and time-depended, the number of viable bacteria were declining with the increasing length of exposure to disinfectants. We conclude that the combination of benzalkonium chloride, DDAC and formic acid, exhibits better activity against *F. novicida* than PHMB at the same concentrations. Interstingly, PHMB has significant activity, against *L. pneumophila, L. pneumophila* grown in amoeba and the *A. polyhgaga* [33]. The primary targets for the PHMB’s disinfectant on the bacterial cell were the outer and the cytoplasmic membranes [34]. PHMB is thought to adhere to and disrupt target cell membranes, causing them to leak potassium ions and other cytosolic components which results in cell death [34]. The 0.2% PHMB was significantly more efficient in killing microorganisms *Enterococcus faecalis*, *Candida albicans* and *Staphylococcus epidermidis* when it was compared with 2.5% sodium hypochlorite and 0.2% chlorhexidine [35]. However, based on the findings of some similar studies, QACs and PHMB are membrane-active agents [36,37] that could cause a loss of structural organization and integrity of the cytoplasmic membrane in the bacteria, together with other damaging effects to the bacterial cell [38]. In our study, the significant increase in the leaked proteins from the treated bacteria confirmed that the bacterial cell membrane was damaged by the disinfectant. Furthermore, the protein leakage from the amoeba-grown bacteria was higher than the protein leakage from the agar-grown bacteria, treated with the disinfectants under the same conditions. We conclude that the bacterial growth in amoeba leads to an increased sensitivity to disinfectants.

In conclusion, among the solutions of the disinfectants prepared in such concentrations, attributed to use in the laboratory, the active substances DDAC and the isopropyl alcohol in the combination were proven to be the best for the destruction of in vitro- and amoeba-grown *F. novicida*, even at a four times lower concentration than the manufacturer had recommended. The obtained results showed that it is possible to inhibit bacterial growth by increasing the concentration of the tested active substance and by extending the contact time. Our findings can have the potential applications in decontamination strategies in areas where the waterborne tularemia cases are frequent. However, a further investigation should be conducted to understand *Francisella’s* susceptibility to disinfectants after being grown in the amoeba.
